# Lung Cancer Clinical Trials with a Seamless Phase II/III Design: Systematic Review

**DOI:** 10.3390/jcm11237176

**Published:** 2022-12-02

**Authors:** Dionysios Palermos, Theodoros N. Sergentanis, Maria Gavriatopoulou, Panagiotis Malandrakis, Theodora Psaltopoulou, Evangelos Terpos, Ioannis Ntanasis-Stathopoulos

**Affiliations:** 1 Department of Clinical Therapeutics, School of Medicine, National and Kapodistrian University of Athens, 11528 Athens, Greece; 2 Department of Public Health Policy, School of Public Health, University of West Attica, 11521 Athens, Greece

**Keywords:** seamless phase II/III, lung cancer, adaptive designs, clinical trial designs

## Abstract

Current lung cancer clinical research focuses on biomarkers and personalized treatment strategies. Adaptive clinical trial designs have gained significant ground due to their increased flexibility, compared to the conventional model of drug development from phase I to phase IV trials. One such adaptive approach is the seamless phase II/III design, which has been used to reduce the total sample size and drug development time. In this context, an algorithmic systematic search was conducted in MEDLINE (PUBMED), SCOPUS, EMBASE and Cochrane Central Register of Controlled Trials until 31 June 2022 in order to identify lung cancer trials of systematic treatments that have employed the seamless phase II/III methodology and to describe their characteristics. The search strategy yielded a total of 1420 records that were screened through their title and abstract; 28 eligible trials were included in the systematic review. Based on the study endpoints, the most common subtype included phase II/III trials with inefficacy/futility analyses (61%; 17/28), followed by dose escalation phase II/III trials (18%; 5/28), one multi-arm multi stage trial and 5 trials with other design (18%). Most eligible trials were open-label (71%; 20/27), included patients with non-small cell lung cancer (82%; 23/28), evaluated targeted therapies and/or immunotherapies (82%; 23/28) and recruited patients with advanced disease (89.3%; 25/28). In conclusion, the seamless phase II/III design is a feasible and suitable approach in lung cancer research, with distinct design subcategories according to study endpoints.

## 1. Introduction

Lung cancer is the most lethal and one of the most commonly diagnosed cancers around the world, with 2.2 million new cases and 1.79 million deaths every year [1]. The two major classifications of lung cancer include small cell lung cancer (SCLC) and non-small cell lung cancer (NSCLC), whereas NSCLC is the most frequent, accounting for 84% of all diagnoses [2]. NSCLC can be further subcategorized into several histologic subtypes, with adenocarcinoma and squamous cell carcinoma being the most common [3]. Despite being less common than NSCLC, SCLC is a very aggressive form of lung cancer, representing about 15% of all diagnoses, and it is classified as limited or extensive, depending on disease staging [4]. Currently, there is an urgent and unmet need for both novel therapeutic choices and prognostic biomarkers, since lung cancer has a remarkably poor prognosis, with the 5-year survival rate reaching 25% for NSCLC and only 7% for SCLC [2].

With the recent addition of novel biomarkers (and the corresponding treatments) for Immunotherapy (IT) (PD-1, PD-L1, CTLA-4) and Targeted Therapy (TT) (EGFR, BRAF, MET mutations/ALK, ROS1, RET, NTRK translocations) to the rapidly-changing lung cancer treatment landscape [5], the conventional model of drug development from phase I to phase II and phase III trials has been challenged due to its evident lack of flexibility, since it generally does not allow for seamless cohort expansion or early termination in response to interim efficacy data [6]. Instead, adaptive clinical trial designs, which allow for prespecified modifications to various aspects of the trial based on prospectively accumulating data, have been gaining significant ground by combining accelerated decision making, improved use of resources, earlier detection of efficacy or futility and easy trial expansion to specific subpopulations of interest [6,7].

Among the various adaptive designs, one that has been consistently used in cancer clinical trials is the seamless phase II/III design, which combines objectives that are regularly achieved through individual Phase II and Phase III trials, with the transition between phases occurring seamlessly, without a pause [8]. Essentially, this design can be used to combine a dose-determination phase II trial with a confirmatory phase III trial (“operationally seamless”) or to use efficacy-related data from the phase II portion in the pivotal phase III portion (“inferentially seamless”), potentially reducing the total sample size needed and the drug development time [9]. Furthermore, phase II/III trials can be subcategorized based on the nature of their primary and secondary endpoints. Specifically, these designs may include “Phase II/III trials with between-arm Phase II analyses”, “Phase II/III trials with multiple experimental arms”, “Phase II/III designs with Overall Survival (OS) as the phase II and III endpoint”, “Phase III with an aggressive inefficacy/futility analysis” [10]. Despite its broad use in lung cancer research, this design is very heterogenous, with several design subcategories that have not been adequately defined in the current literature. In this context, this systematic review aims to critically discuss the methodology and the outcomes of lung cancer clinical trials of systematic treatments with a seamless phase II/III design.

## 2. Materials and Methods

This systematic review was designed and performed according to the Preferred Reporting Items for Systematic Reviews and Meta-Analyses (PRISMA) guidelines [11]. The systematic search was conducted in MEDLINE (PUBMED), SCOPUS, EMBASE and Cochrane Central Register of Controlled Trials from inception to June 30th, 2022. The algorithm that was used for the search process was as follows: “(Seamless OR “phase 2/3” OR “phase 2,3” OR “phase II/III” OR “phase 2b/3” OR “phase IIb/III”) AND ((lung AND (cancer OR cancers OR carcinoma OR carcinomas OR malignant OR malignancy OR malignancies OR neoplasm OR neoplasms)) OR NSCLC OR SCLC)”. Additionally, to improve the validity of the search process, the snowball technique (reference screening for eligible studies) was performed on the reviews, systematic reviews and/or meta-analyses found in the aforementioned databases. Duplicate records were excluded and all required data were recorded on an Excel spreadsheet. Due to the nature of the research question, a meta-analysis was not performed, however we calculated the median projected enrollment and median duration of eligible trials with available data.

### 2.1. Study Eligibility Criteria

Detailed eligibility criteria are described in Table 1 (PICO framework) [12]. Eligible studies had to include patients with lung cancer (NSCLC or SCLC) treated with any systematic treatment and implement a prespecified seamless Phase II/III strategy, irrespective of the chosen outcomes. Peer-reviewed publications, conference abstracts, and clinical registry records of ongoing, terminated or completed trials were included in this systematic review, while the utilization of a seamless Phase II/III and use of any systematic treatment should have been mentioned in the title, abstract or full text of the respective document. Studies pertaining exclusively to radiotherapy and/or surgery without referring to chemotherapy, immunotherapy and/or targeted therapy for lung cancer were deemed ineligible. Non-English language studies were excluded. All identified abstracts were then independently identified and reviewed by two reviewers (DP and INS), followed by retrieval and assessment of potentially relevant studies. A third investigator (TS) was responsible for resolving any discrepancies in the study selection process.

### 2.2. Data Extraction

The following data were extracted from the eligible studies: Title, countries and trial sites, condition (NSCLC or SCLC), histology, stage and Eastern Cooperative Oncology Group Performance Status (ECOG-PS) of trials subjects, type of treatment, number of arms, projected and actual enrollment, demographic characteristics of patients (median age, sex), utilization of randomization and/or blinding, stratification factors, statistical power, type of phase II/III design, reason for proceeding to phase III or terminating the trial, primary and secondary endpoints, number of interim analyses, primary endpoint result. All data were recorded in an Excel spreadsheet and presented in tables.

### 2.3. Risk of Bias Assessment

We used the Version 2 of the Cochrane risk-of-bias tool for randomized trials [13] to assess the internal validity of completed trials that published full-text results, because only these trials were providing all data needed to be adequately assessed. The updated version contains five bias domains (D): D1: bias due to the randomization process; D2: bias due to deviations from intended interventions; D3: bias due to missing outcome data; D4: bias related to the outcome measurement; and D5: bias arising from the selection of the reported result. We performed the assessment by answering to a number of signaling questions in each of the five domains and relied on the tool’s algorithm for the final result.

## 3. Results

**Figure 1:**
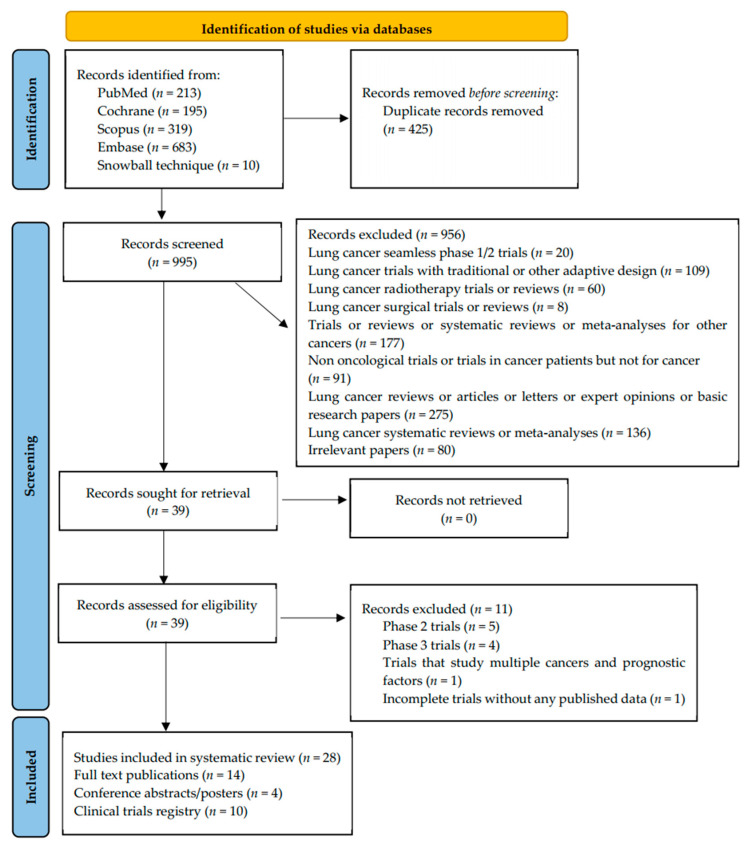
Modified PRISMA_2020 flow diagram of the systematic review.

Our systematic search resulted in identifying 213 records from MEDLINE (PUBMED), 319 records from SCOPUS, 683 records from EMBASE, 195 records from Cochrane Central Register of Controlled Trials, and 10 records from the snowball technique, yielding a total of 1420 records. After excluding all duplicates (*n* = 425), we proceeded to screening (titles and abstracts) of 994 records, of which 956 were excluded due to the following reasons: lung cancer seamless phase 1/2 trials (*n* = 20), lung cancer trials with conventional or other adaptive design (*n* = 109), lung cancer radiotherapy trials or reviews (*n* = 60), lung cancer surgical trials or reviews (*n* = 8), trials or reviews or systematic reviews or meta-analyses for other cancers (*n* = 177), non-oncological trials or trials in cancer patients but not for cancer (*n* = 91), lung cancer reviews or articles or letters or expert opinions or basic research papers (*n* = 275), lung cancer systematic reviews or meta-analyses (*n* = 80), irrelevant papers (*n* = 171). Subsequently, we proceeded to the full-text examination of the remaining 39 records, of which 11 were excluded because of the following reasons: phase 2—only trials (*n* = 5), phase 3—only trials (*n* = 4), trials that study multiple cancers and prognostic factors (*n* = 1), incomplete trials without any published data (*n* = 1). The modified PRISMA 2020 flow diagram is presented in Figure 1.

Finally, we ended up with 28 eligible trials, of which 7 (25%) were completed, 4 (14%) had only completed the phase II portion, 5 (18%) were terminated, 2 (7%) were amended to a phase II design, and 10 (36%) were ongoing. The main characteristics of these trials are presented in Table 2, Table 3, Table 4 and Table 5.

Among trials with available data, the majority (71%; 20/27) were unblinded (open-label), while the most usual reasons for not completing the trial according to its original design (termination/amendment to phase II) were unsatisfactory interim efficacy/safety results (67%; 6/9) and simultaneous approval of competing treatments (33%; 3/9). Most trials were multicenter (96%; 25/26), with median [interquartile range (IQR)] phase III projected enrollment being 460 (400) patients, and median (IQR) duration among completed trials with available data being 49 (27) months. The majority of the trials (82%; 23/28) were recruiting patients with NSCLC, with only 18% (5/28) recruiting patients with SCLC.

### 3.1. Subtypes of Seamless Phase II/III Design

Since the main objective of this systematic review was to focus on the methodology of seamless phase II/III design, we categorized the 28 eligible trials into 4 distinct categories: (a) Phase II/III trials with inefficacy/futility analyses [14,15,16,17,18,19,20,21,22,23,24,25,26,27,28,29,30], (b) Dose escalation Phase II/III trials [31,32,33,34,35], (c) Multi-Arm Multi Stage (MAMS) phase II/III trials [36], (d) Trials with other design [37,38,39,40,41].

(a) Phase II/III trials with inefficacy/futility analyses (Inferentially Seamless)

Among the 28 eligible trials, this design subtype was the most common, accounting for more than half of the included studies (61%; 17/28). The trials in this subgroup were characterized by homogeneity regarding endpoints, with 94% (16/17) having overall survival (OS) or progression-free survival (PFS) as the primary endpoint of the phase III portion. The only aspect that varied significantly among trials was the primary objective of the phase II portion. More specifically, the primary outcome of the phase II portion was a response-related endpoint [overall response rate (ORR), response rate (RR)] for 41% (7/17) of the trials, and a survival-related endpoint (OS, PFS) for 47% (8/17), whereas 1 trial (6%; 1/17) had both RR and PFS as primary outcomes of the phase II portion.

With respect to patient and disease characteristics, these trials recruited mainly patients with NSCLC (88%; 15/17), with stage IIIB-IV or stage IV disease (93%; 14/15), whereas no studies recruited patients with ECOG PS > 2. Most trials with available demographic data (78%; 7/9) recruited mainly male patients (ranging from 54% to 73%), with just two trials recruiting more female than male patients. The median patient age ranged from 58.5 to 75 years. Table 2 shows the main characteristics of these trials, with additional trial characteristics featured in Tables S1–S6.

(b) Dose escalation Phase II/III trials (Operationally Seamless)

This was the second most common subtype, which was applied in 18% (5/28) of the eligible trials (Table 2). In this particular design, the phase II portion aimed to determine the optimal dose of the investigation product, by examining the risk-benefit ratio between dose limiting toxicities and interim efficacy results. Interestingly, OS was the primary endpoint for the phase III portion of most trials (4/5; 80%), followed by PFS in one trial.

In terms of patient and disease characteristics, this was the preferred design for the majority of SCLC trials (60%; 3/5), with all of them recruiting patients with ECOG PS < 2. Among trials with available data, enrollment mainly pertained to male patients, with the respective proportion ranging from 61.6% to 75.8%, while their median age was above 60 years old, ranging from 61.6 to 62.6 years. The main characteristics of these trials are provided in Table 3, with additional trial characteristics featured in Tables S7–S9.

(c) Multi-Arm Multi Stage (MAMS) phase II/III trials

This design was used by only one trial [36], which is an ongoing multi-cohort study with 9 arms evaluating multiple targeted therapies and immunotherapies for NSCLC, according to their respective mutational biomarker, as identified by two novel blood-based next-generation sequencing (NGS) circulating assays (Table 4). This trial contains both phase II and phase III sub-studies, with distinct primary outcomes. More specifically, response-related endpoints (e.g., ORR) correspond to the phase II portions, whereas the phase III portions are characterized by survival-related endpoints (e.g., OS, PFS). This trial was initiated in 2017 and is currently enrolling patients with stage IIB-IV NSCLC and ECOG PS ≤ 2. Additional trial characteristics are provided in Tables S10 and S11.

(d) Trials with other design

The remaining trials (18%; 5/28) could not be categorized into one of the abovementioned groups for different reasons (Table 5). Two of these trials did not report the exact phase II/III design [40,41] but only mentioned the term “phase II/III” in the title without further explaining the methodology. Two other trials shared the same design, in which the phase II and phase III primary endpoints were feasibility/compliance and DFS (disease-free survival), respectively. Feasibility/compliance was defined as adherence to a specific regimen for a prespecified time period, by means of self-reporting or pill counting [37,38]. The last trial [39] reported a design in which patients stayed on trial even after disease progression, to capture and evaluate the possible chemo-sensitization effects seen in previous trials with the same investigational medicinal product. More information on these trials is expected in the near future with the full-text publications becoming available. Additional trial characteristics are featured in Tables S12 and S13.

### 3.2. Risk of Bias Assessment

**Figure 2:**
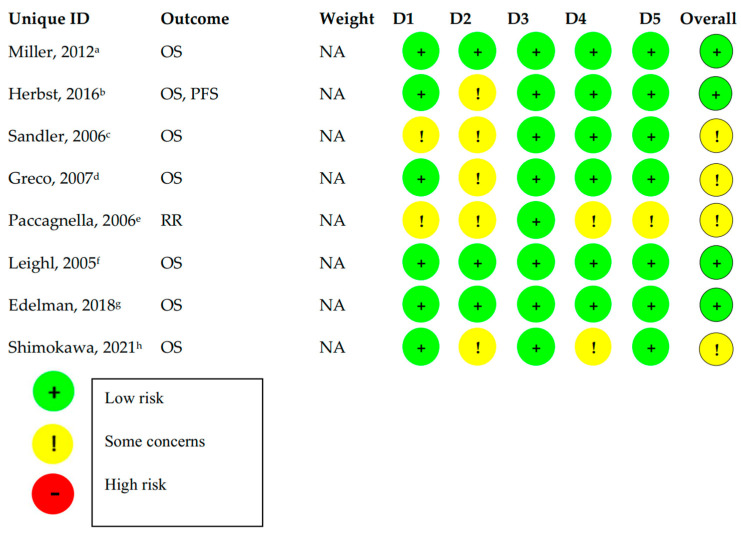
Risk of Bias Assessment on all completed trials using the Cochrane risk-of-bias tool for randomized trials (RoB 2). OS: overall survival; PFS: progression-free survival; RR: response rate; NA: not applicable; ^a^ [14], ^b^ [31], ^c^ [15], ^d^ [18], ^e^ [16], ^f^ [23], ^g^ [32], ^h^ [17].

We were able to perform a risk of bias assessment only on the 8 completed trials that presented full text results (Figure 2). For this reason, we used the Cochrane risk-of-bias tool for randomized trials (RoB 2). The results of this process are presented in Figure 2. Among these trials, 50% (4/8) were assessed to have ‘some concerns’ for bias, mainly arising from not being blinded (open-label), which could lead to deviations from the intended interventions and/or biased measurement of the outcome. The other four trials were assessed to have a ‘low risk’ for bias. The detailed assessment is shown in Table S14.

## 4. Discussion

Improving survival and quality of life of patients with lung cancer still remains challenging due to several factors, including late detection and rapid disease progression [2]. Because of its substantial metastatic potential and low survival rate, advanced NSCLC and SCLC are particularly hard to treat [42], creating a large unmet need for effective novel therapies. In this setting, the seamless phase II/III clinical trial design seems to be appropriate [43], offering potentially shortened drug development time and significant treatment flexibility, as it can be incorporated into multiple-arm trials. Our results are in accordance with this viewpoint, since the vast majority of eligible trials (89.3%; 25/28) were recruiting patients with stage III/IV NSCLC or SCLC. Additionally, the current era of oncological clinical research is defined by the emergence of personalized medicine, with biomarkers used to identify subpopulations of patients who are most likely to benefit from targeted therapies and immunotherapies [5]. This landscape favors seamless drug development, by facilitating the whole process with rapid cohort expansions and less bureaucratic hurdles [44,45]. Our results confirm this perspective, with 82% (23/28) of eligible trials testing targeted therapies, immunotherapies or combinations of both. The seamless approach may be also preferable in terms of protecting patients from ineffective therapies, owing to the rigorous interim analyses that are being performed to detect early futility [46].

Conversely, by implementing the seamless phase II/III design, the sponsor *de facto* commits to the conduct of a phase III trial, which entails certain logistical and methodological challenges. Firstly, the phase III infrastructure has to be already in place by securing substantial patient and financial resources, thus increasing the investment’s total risk. At the same time, the potential commercial approval of a competing treatment during a phase II/III trial complicates its course significantly, which does not occur with standalone phase II trials [10]. We observed this particular issue in three different trials that had to be terminated or amended to phase II, in response to the approval of PD-1 (programmed cell death-1) inhibitors (pembrolizumab, nivolumab) for the treatment of advanced NSCLC [20,24,25]. Finally, the seamless phase II/III design might be susceptible to “operational bias”, which occurs when multiple trial adaptations are deemed necessary after the evaluation of interim unblinded data [47]. We also noticed this issue in our systematic review, with the majority of evaluable trials (71.4%; 20/27) being open-label, a trend that was more pronounced in terminated/amended trials (86%; 6/7). However, this issue may also apply to conventional phase III trials, since in both designs the trial analysts will be unblinded to confidential interim results [46]. Therefore, pivotal seamless studies should be implemented when substantial data from prior pre-clinical and phase I trials already exist. Furthermore, they should clearly prespecify the optimal level of clinical efficacy and, most importantly, they should clearly define the role of all stakeholders, to avoid bias and ensure trial integrity [45].

Several previous publications [48,49,50] have described the biostatistical background and general characteristics of the seamless phase II/III trial design, but, to the best of our knowledge, this is the first systematic review about lung cancer trials with a seamless phase II/III design. Furthermore, despite the variability in terminology regarding the specific adaptive trial design, we believe that our systematic search was rigorous enough to provide a comprehensive view of all available data in the field. By systematically searching the major databases, we managed to track eligible trials and to extract all relevant data, while simultaneously any potential selection bias was minimized [51]. An important limitation of this systematic review is the missing data during data abstraction, regarding mainly ongoing trials and trials that were presented only as conference abstracts not reporting all information about study design or patient characteristics. However, this issue may be rectified when the full-text publications become available. Finally, another limitation may pertain to the exclusion of non-English studies.

## 5. Conclusions

As biomarker assessment becomes an established practice in cancer care and emerging treatments continuously enter into clinical trials, the conventional model of discrete trial phases is currently being replaced by adaptive trial designs, which offer increased flexibility and improved use of resources. In particular, the seamless phase II/III design is being consistently used in lung cancer research for novel chemotherapies, immunotherapies and targeted therapies with distinct design subcategories according to study endpoints. However, more effort should be made to educate all stakeholders about its advantages and disadvantages, in order to optimize clinical trial design and conduct as well as patient outcomes.

## Figures and Tables

**Table 1:** Summary of PICOS (population, intervention, comparator, outcomes, study design) criteria.

Category	Inclusion Criteria
Population	Lung cancer patients (NSCLC or SCLC)
Interventions	Administration of any systematic treatment (chemotherapy, immunotherapy, targeted therapy)
Comparator	Administration of any systematic treatment (chemotherapy, immunotherapy, targeted therapy)
Outcomes	No restrictions
Study design	Seamless Phase II/III trials were defined as having both a prespecified Phase II (exploratory) and Phase III (confirmatory) portion that were occurring seamlessly, i.e., without a pause.
Other	Peer-reviewed publications, conference abstracts or Clinical registry records in the English language

**Table 2:** Phase II/III trials with aggressive inefficacy/futility analyses.

Reference	Status	Condition	Treatment	No Arms	Year Started	Enrollment (Projected/ Actual)	Phase 3 Randomisation	Blinding	Phase II/III Type	Primary Endpoints	Secondary Endpoints	No Interim Analyses
[14]	Completed	NSCLC	TT ^a^ (TKI ^k^)	2	2008	560/585	Yes	Triple ^f^	Phase IIb analysis (ORR) before full phase III accrual	OS	PFS, ORR, DOR, safety,	1
[15]	Completed	NSCLC	Combination of IT ^b^ (VEGF ^l^ inhibitor) and CT ^c^	2	2002	Originally: 640 In January 2004: 842/878	Yes	Open label	Phase II with 2 interim analyses with stopping rules for efficacy and futility (OS, PFS)	OS	PFS, RR	2
[16]	Completed	NSCLC	CT	2	1998	324/324	Yes	NR	Phase II trial (RR)amended to further evaluate the impact of the two CT regimens on OS	OS, RR	Toxicity	NR
[17]	Completed	SCLC	CT	2	2013	250/258	Yes	Open label	Phase II (ORR) to assess adequate efficacy in elderly patients	Phase II:ORR Phase III: OS	PFS, toxicity	2
[18]	Completed	NSCLC	CT	6	2004	330/337	Yes	Open label	4-arm prospective randomized phase II trial (RR) extended to a randomized 2-arm phase III	OS, RR	PFS, toxicity	1
[19]	Completed only phase II portion	NSCLC	Combination of TT (TKI) and CT	2	2005	750/296	Yes	Double-blind	Phase II (PFS, RR, toxicity) which would continue to full phase III (OS) accrual if the HR for PFS was 0.77 with no toxicity concerns	Phase II: PFS, RR, toxicity Phase III: OS	Health economics, tissue markers, QoL ^g^	1
[20]	Completed only phase II portion	NSCLC	Combination of IT (vaccine) and CT	2	2012	NR/222	Yes	Double-blind	Phase IIB (PFS) to validate the TrPAL biomarker; Phase III (OS)	PFS	OR, DoR ^h^, OS, safety, time to OR	1
[21]	Terminated	NSCLC	TT (TKI)	3	2014	100 for phase II, 500 for phase III/100	Yes	Open Label	Data from the Phase II part will determine the sample size in the Phase III part	PFS	OS, ORR, DoR, QoL, safety, PK ^i^	1 every 3–6 months
[22]	Terminated	NSCLC	Combination of IT (interferons) and CT	2	1980s	46/37	Yes	Open Label	Embedded phase II (RR) trial within the combination arm	ORR	Safety	1
[23]	Terminated	NSCLC	Combination of a MMPI ^d^ with CT	2	2000	750/774	Yes	Triple ^f^	Phase II (RR, toxicity), phase III (OS)	OS	PFS, RR, DoR, QoL, toxicity	1
[24]	Amended to phase II	NSCLC	TT (TKI) vs. CT	3	2014	400/98	Yes	Open label	Phase II (PFS), Phase III (OS)	PFS, OS	DoR, toxicity	1
[25]	Amended to phase II	NSCLC	IT (PD-L1 inhibitor) vs. CT	2	2014	NR/53	Yes	Open label	Phase II/III trial (OS, PFS)	OS, PFS	(IA-PFS), OS, toxicity	NR
[26]	Ongoing	SCLC	Combination of IT (PD-L1 inhibitor) and CRT ^e^	2	2019	506/N/A	Yes	Open label	Phase II (PFS), Phase III (OS)	Phase II: PFS Phase III: OS	PFS, ORR, DMFS ^m^, QoL, TMB ^j^	NR
[27]	Ongoing	NSCLC	IT (PD-1 inhibitor) and CT	2	2020	700/NR	Yes	Phase II: Open label Phase III: Participant	Phase II(ORR, adverse events) to proceed to phase III (PFS)	Phase II: ORR, AEs Phase III: PFS	Phase II: PFS, DoR, OS Phase III: ORR, DoR, OS	NR
[28]	Ongoing	NSCLC	Combination of IT (PD-1 inhibitor) and CT	2	2021	100/NR	Yes	Open label	Phase II (PFS), Phase III (OS)	Phase II: PFS; Phase III: OS	ORR, QoL, AEs	NR
[29]	Ongoing	NSCLC	Combination of IT (PD-1 inhibitor) and CT	2	2022	286/NR	Yes	Open label	Only patients with disease control at 6 months (phase II) will be randomized 1:1 (phase III)	Phase II (OS), phase III (OS)	AEs, PFS, QoL	0
[30]	Terminated	NSCLC	Combination of IT (CD-20 inhibitor) and TT	2	2014	NR/9	Yes	Open Label	Phase II: PFS Phase III: OS	PFS, OS	Safety	NR

^a^ Targeted therapy, ^b^ Immunotherapy, ^c^ Chemotherapy, ^d^ Matrix metalloproteinase inhibitor, ^e^ Chemoradiotherapy, ^f^ Participants, Investigators, Sponsor, ^g^ Quality of Life, ^h^ Duration of Response, ^i^ Pharmac.okinetics, ^j^ Tumour Mutational Burden, ^k^ Tyrosine Kinase Inhibitor, ^l^ Vascular Endothelial Growth Factor, ^m^ Distant Metastasis-Free Survival. OS: overall survival; PFS: progression-free survival; NR: not reported; AEs: adverse events.

**Table 3:** Dose escalation Phase II/III trials.

Author_Year	Status	Condition	Treatment	No Arms	Year Started	Enrollment (Projected/Actual)	Phase 3 Randomisation	Blinding	Phase II/III Type	Primary Endpoints	Secondary Endpoints	No Interim Analyses
[31]	Completed	NSCLC	IT (PD-1 inhibitor)	3	2013	920/1034	Yes	Study: Blinded study statisticianPD-L1 positivity: double-blindedPFS: independent radiologist.	Multiple dose phase II (ORR,OS) to proceed to phase III (OS)	OS, PFS, Safety	ORR, DoR	2
[32]	Completed	SCLC	Combination of IT (glycolipid GD2 inhibitor)and CT	3	2017	460/483	Yes	Open label	Phase II: intra-subject dose escalation. Phase III: OS	OS	PFS, ORR, CBR ^a^	0
[33]	Ongoing	SCLC	CT	2	2018	480/NR	Yes	Open label	Phase II: Dose determination Phase III: Randomized, efficacy study	Phase II: Safety, Optimal dosePhase III: OS	PFS, ORR, QoL	1
[34]	Ongoing	SCLC	Combination of TT (TKI)and CT	2	2019	313/NR	Yes	Double-blind	Phase II: Dose findingPhase III: PFS	Phase II: Adverse events Phase III: PFS	NR	NR
[35]	Ongoing	NSCLC	IT (PD-L1/CTLA-4 bispecific inhibitor) and TT (TKI)	2	2021	522/NR	Yes	Open Label	Phase II: DLTs Phase III: OS, PFS	Phase II: DLTsPhase III: OS, PFS	ORR, DCR ^b^, DoR, CBR, TTR ^c^	NR

^a^ Clinical Benefit Rate, ^b^ Disease Control Rate, ^c^ Time to Response. IT: immunotherapy; CT: chemotherapy; TT: targeted therapy; TKI: tyrosine kinase inhibitor; OS: overall survival; PFS: progression-free survival; NR: not reported.

**Table 4:** Multi-Arm Multi Stage (MAMS) phase II/III trials.

Status	Condition	Treatment	No Arms	Year Started	Enrollment (Projected/Actual)	Phase 3 Randomisation	Blinding	Phase II/III Type
Ongoing	NSCLC	Multiple TTs and ITs	9	2017	700/NR	Yes	Open label	MAMS

IT: immunotherapy; CT: chemotherapy; TT: targeted therapy; TKI: tyrosine kinase inhibitor; NR: not reported; NSCLC: non-small cell lung cancer; MAMS: multi-arm multi stage.

**Table 5:** Trials with other study designs.

Reference	Status	Condition	Treatment	Νο Arms	Year Started	Enrollment (Projected/ Actual)	Phase 3 Randomisation	Blinding	Phase II/III Type	Primary Endpoints	Secondary Endpoints	Nο of Interim Analyses
[37]	Completed only phase II portion	NSCLC	Combination of TT (TKI) and CT	4	2009	164/150	Yes	Open Label	Phase II adjuvant trial (feasibility) Phase III: DFS	Feasibility defined as 80% of patients being able to start adjuvant therapy within 2 months after surgery	tolerability, compliance biomarker distribution	0
[38]	Completed only phase II portion	NSCLC	TT (TKI)	2	2008	112/142	Yes	Quadruple ^b^	Phase II (compliance/feasibility of regime), phase III (DFS)	Compliance based on both self-reporting and pill counts. Patients were classified as compliant if they received treatment of at least 12 weeks	OS, recurrence-free survival ^a^, toxicity, QOL	1
[39]	Terminated	NSCLC	Combination ofIT (vaccine) and CT	3	2013	240/135	Yes	Open Label	Design with patients staying on trial after progression	OS	RR	NR
[40]	Ongoing	NSCLC	TT (TKI)	2		300/492	Yes	Open label	NR	PFS assessed by blinded independent radiologist	IA-PFS ^c^ IC-PFS, EC-PFS, ORR, DoR, OS, QoL, DCR	NR
[41]	Ongoing	NSCLC	TT (TKI)	2	2019	360/362	Yes	Open label	NR	PFS	ORR, DCR, iORR ^d^, IC-PFS OS, DoR, safety	NR

^a^ Relapse Free Survival, ^b^ Participant, Care Provider, Investigator, Outcomes Assessor, ^c^ Investigator Assessed, ^d^ Intracranial objective response rate. IT: immunotherapy; CT: chemotherapy; TT: targeted therapy; TKI: tyrosine kinase in-hibitor; NR: not reported; NSCLC: non small cell lung cancer; DFS: disease-free survival; OS: overall survival; RR: response rate; QOL: quality of life; DoR: duration of response; DCR: disease control rate.

## Data Availability

Data available upon request from authors.

## Supplementary Materials

The following supporting information can be downloaded at: https://www.mdpi.com/article/10.3390/jcm11237176/s1, Table S1: Phase II/III trials with aggressive inefficacy/futility analyses (Additional characteristics); Table S2: Phase II/III trials with aggressive inefficacy/futility analyses (Additional characteristics); Table S3: Phase II/III trials with aggressive inefficacy/futility analyses (Additional characteristics); Table S4: Phase II/III trials with aggressive inefficacy/futility analyses (Additional characteristics); Table S5: Phase II/III trials with aggressive inefficacy/futility analyses (Additional characteristics); Table S6: Phase II/III trials with aggressive inefficacy/futility analyses (Additional characteristics); Table S7: Dose escalation Phase II/III trials (Additional characteristics); Table S8: Dose escalation Phase II/III trials (Additional characteristics); Table S9: Dose escalation Phase II/III trials (Additional characteristics); Table S10: Multi-Arm Multi Stage (MAMS) phase II/III trials (Additional characteristics); Table S11: Multi-Arm Multi Stage (MAMS) phase II/III trials (Additional characteristics); Table S12: Trials with other design (Additional characteristics); Table S13: Trials with other design (Additional characteristics); Table S14: Detailed Cochrane risk-of-bias tool for randomized trials (RoB 2) assessment.
