# Supplementary material for: Lung Cancer Clinical Trials with a Seamless Phase II/III Design: Systematic Review
Source: J Clin Med. 2022 Dec 2;11(23):7176. doi: 10.3390/jcm11237176 (PMC9739886; doi:10.3390/jcm11237176)
Supplement: Supplementary file 1 [file jcm-11-07176-s001.zip › jcm-2025759-supplementary.pdf]

Table S1: Phase II/III trials with aggressive inefficacy/futility analyses (Additional characteristics)

1st author or ClinicalTrials.gov Identifier	Status	Countries	Centres	Histology	Stage	PS (Performance Status)	Median age (years)	Percentage males	Duration (Months)
Miller	Completed	15	86	Adenocarcinoma	IIIB, IV	ECOG 0-2	58.5	40.5%	66 (April 2008-October 2013)
Sandler	Completed	3	108	All types except squamous cell carcinoma	IIIB, IV	ECOG 0-1	43% ≥ 65 years old	0,54	49 (August 2002-September 2006)
Paccagnella	Completed	NR	NR	All types	IIIB, IV	ECOG 0-2	61.5	0,78	NR
Shimokawa	Completed	1	34	All types	IIIB, IV	ECOG 0-2	62	0,59	39 (June 2004-September 2007)
Greco	Completed only phase II portion	6	20	Adenocarcinoma, squamous	IIIB,IV	ECOG 0-1	59	0,585	88 (September 2005-Jan 2013)
Goss	Completed only phase II portion	9	45	All types	IV	ECOG 0-1	61	0,64	51 (April 2012-July 2016)
Quoix	Terminated	7	74	All types	I-IV	ECOG 0-1	65	0,33	31 (November 2014-June 2017)
Camidge	Terminated	NR	NR	Adenocarcinoma, squamous cell, large cell	III-IV	ECOG 0-2	61.75	70.27%	NR
Schiller	Terminated	14	63	All types	IIIB, IV	ECOG 0-2	61,3	0,73	106 (April 2000-February 2009)

Table S2: Phase II/III trials with aggressive inefficacy/futility analyses (Additional characteristics)

1st author or ClinicalTrials.gov Identifier	Status	Countries	Centres	Histology	Stage	PS (Performance Status)	Median age (years)	Percentage males	Duration (Months)
Leighl	Amended from phase II/III to phase II	1	NR	Squamous cell carcinoma	III, IV	ECOG 0-2	67.3	66.6%	57 (Sep 2014-June 2019)
Edelman	Amended from phase II/III to phase II	1	NR	Squamous cell carcinoma	IIIB,IV	ECOG 0-2	68.5	63.5%	NR
Borghaei	Ongoing	1	38	N/A	NR	ECOG 0-2	75	N/A	N/A
NCT03811002	Ongoing	2	526	N/A	Limited	ECOG 0-2	N/A	N/A	N/A
NCT04750083	Ongoing	1	10	Nonsquamous	IV	ECOG 0-1	N/A	N/A	N/A
NCT04929041	Ongoing	1	15	All types	IV (Patients with Stage IIIB and IIIC disease eligible if they are not a candidate for combined chemotherapy and radiation	ECOG 0-2	N/A	N/A	N/A
NCT05255302	Ongoing	1	37	All types	IV	ECOG 0-1	N/A	N/A	N/A
NCT02926638	Ongoing	1	37	All types	IV	ECOG 0-1	N/A	N/A	N/A

Table S3: Phase II/III trials with aggressive inefficacy/futility analyses (Additional characteristics)							
1st author or ClinicalTrials.gov Identifier	Stratification factors	Statistical power for phase III	Reason for proceeding to phase III	Reason for not proceeding to phase III	Reason for trial termination	Reason for amendment	Primary endpoint result
Miller	Sex, ECOG PS (0–1 vs 2)	560 patients with 359 events to have a 90% power at the one-sided 0·025 significance level to reject the null hypothesis with a HR of 0·70	≥3 responses in the first 40 afatinib-treated patients (10)	-	-	-	HR=1.08, 95% CI 0.86–1.35; one-sided p=0.74
Sandler	Measurable versus non-measurable disease, prior radiation therapy versus no prior radiation therapy, prior weight loss of less than 5% versus 5% or more,, stage IIIB with pleural effusion versus stage IV or recurrent disease	80.5% power to detect a HR for death of 0.80 in the group treated with chemotherapy plus bevacizumab with an overall type I error of 2.5%	Recommendation by the data monitoring committee and the ECOG lung committee	-	-	-	HR for death: 0.79; 95% CI, 0.67 to 0.92; p=0.003)
Paccagnella	Study site,stage of disease (IIIB vs IV)	324 patients to detect a minimal HR of 1.33 with a power of 0.8 and an error of two-sided of 0.05.	NR	-	-	-	HR= 1.31 (CI, 1.02 to 1.68, p=0 .044)
Shimokawa	Sex, ECOG PS 0/1 versus 2, and stage of disease (IIIB vs. IV)	With a type I error of 5% (2-sided) and a type II error of 20%, a minimum of 330 patients was calculated to be sufficient	The phase II portion indicated the triplet PCG and the doublet of gemcitabine/vinorelbine as the 2 best regimens on the basis of RR	-	-	-	Median survival in the PCG arm was 10.3 months and was 10.7 months in the gemcitabine/vinorelbine arm (P = 0.269)

Table S4: Phase II/III trials with aggressive inefficacy/futility analyses (Additional characteristics)

1st author or ClinicalTrials.gov Identifier	Stratification factors	Statistical power for phase III	Reason for proceeding to phase III	Reason for not proceeding to phase III	Reason for trial termination	Reason for amendment	Primary endpoint result
Greco	gender, participating center, disease stage (IIIB vs IV), weight loss (≥ 5% vs < 5%), prior adjuvant chemotherapy (yes vs no)	522 patients,with a one-sided alpha of 2.5% and 90% power to detect a 25% risk reduction with the use of cediranib, equivalent to a 2.8-month improvement in OS	-	Due to imbalances in assigned causes of death	-	-	The complete and partial RR was 38% (duration, 4.4 months) for cediranib patients and 16% for placebo patients (P< .001).<br>HR for PFS= 0.74 (95% CI, 0.53- 1.04; p=0.08)
Goss	Baseline value of TrPAL (≤ or > the upper limit of normal [ULN]), chemotherapy regimen (cisplatin-based or carboplatin-based),<br>histology, addition or not of bevacizumab, ECOG PS (0 or 1), centre	Statistical power of 92% with 151 PFS events	-	Due to the rapid evolving treatment landscape with the development of pembrolizumab and nivolumab	-	-	HR= 0.74 (95% CI 0.55–0.98); one-sided p=0.019)<br>HR for patients with TrPAL ≤ULN=0.75 (95% CI 0.54–1.03);<br>HR for patients with TrPAL>ULN=0.77 (95% CI 0.42–1.40)
Quoix	Sensitizing EGFR mutation (L858R, del19, or other), territory of residence at time of randomization (Asia or non-Asia)	A total of 640 PFS events will provide approximately 80% power to detect a HR of 0.80 at a 0.025 (1-sided) significance level	-	-	Lack of efficacy for IMP (lower confirmed response rates than anticipated based on preliminary data)	-	NR

Table S5: Phase II/III trials with aggressive inefficacy/futility analyses (Additional characteristics)							
1st author or ClinicalTrials.gov Identifier	Stratification factors	Statistical power for phase III	Reason for proceeding to phase III	Reason for not proceeding to phase III	Reason for trial termination	Reason for amendment	Primary endpoint result
Camidge	Histology	Approximately 88% for comparing response rates of 15% and 50% using a two-sided 0.05 significance level			The number of responses did not provide further supporting evidence of the efficacy of the treatment	-	Three of 18 patients (17%) in the chemotherapy arm and two of 18 patients (11%) in the combination arm had partial responses to therapy (p =0.75) (95% CIs: 3.6%-41.4% for the chemotherapy arm and 1.4%-34.7% for the combined arm).
Schiller	stage (IIIB v IV), PS (0 and 1 vs 2), center	750 patients (581 deaths) to have 90% power to detect a 2.8-month difference (25% reduction in risk of death)			Interim safety analysis revealed no survival advantage and increased toxicity in the experimental arm	-	Median OS was 8.6 months for BMS-275291 versus 9.2 months for placebo (p=0.030)
Leighl	NR	NR	-	-	-	Due to the rapid development and approval of immunotherapy in NSCLC	Two confirmed partial responses observed [ORR = 6% (95% CI: 0%-.15%)]
Edelman	PS (0-1 vs. 2) sex (male vs. female), smoking status (current vs. former/never)	NR			-	Due to the approval of nivolumab in the second line setting in NSCLC	11 responses among the 68 eligible and analyzable patients on the durvalumab arm (ORR= 16% [95% CI 7-25%]
Borghaei	institution, ECOG performance status (0,1 vs 2), sex (male vs female).	183 patients per arm with a one-sided alpha level of 5% and a power of 80%	-	-	-	-	NR

Table S6: Phase II/III trials with aggressive inefficacy/futility analyses (Additional characteristics)							
1st author or ClinicalTrials.gov Identifier	Stratification factors	Statistical power for phase III	Reason for proceeding to phase III	Reason for not proceeding to phase III	Reason for trial termination	Reason for amendment	Primary endpoint result
NCT03811002	NR	NR	-	-	-	-	N/A
NCT04750083	NR	NR	-	-	-	-	N/A
NCT04929041	NR	NR	-	-	-	-	N/A
NCT05255302	PS (0 versus 1), histology (SCC versus non-SCC), PD-L1 (PD-L1 < 1% versus 49%≥PD-L1 ≥ 1% versus PD-L1>49%), sex and response at randomization (partial response versus stabilisation)	NR	-	-	-	-	N/A
NCT02926638	PS (0 versus 1), histology (SCC versus non-SCC), PD-L1 (PD-L1 < 1% versus 49%≥PD-L1 ≥ 1% versus PD-L1>49%),	NR	-	-	-	-	N/A

Table S7: Dose escalation Phase II/III trials (Additional characteristics)

1st author or ClinicalTrials.gov Identifier	Status	Countries	Centres	Histology	Stage	PS (Performance Status)	Median age (years)	Percentage males	Duration (Months)
Herbst	Completed	24	202	All types	IIIB, IV	ECOG 0-1	62.6	61.6%	85 (August 2013-September 2020)
Edelman	Completed	22	198	N/A	Limited Extensive	ECOG 0-1	61.6	75.8%	33 (June 2017-March 2020)
Paz-Ares L	Ongoing	18	119	N/A	NR	ECOG 0-1	N/A	N/A	N/A
NCT04254471	Ongoing	1	1	N/A	Extensive	ECOG 0-1	N/A	N/A	N/A
NCT05001724	Ongoing	1	NR	NR	NR	ECOG 0-1	N/A	N/A	N/A

Table S8: Dose escalation Phase II/III trials (Additional characteristics)

1st author or ClinicalTrials.gov Identifier	Stratification factors	Statistical power for phase III	Reason for proceeding to phase III	Reason for not proceeding to phase III	Reason for trial termination	Reason for amendment	Primary endpoint result
Herbst	ECOG PS (0 vs 1), region (East Asia vs not East Asia), extent of PD-L1 expression (tumour proportion score ≥50% vs 1–49%)	estimation of 920 enrolled patients,550 patients deaths to achieve an 80% power to detect a HR of 0.70 for overall survival in the total population	Full phase III accrual was decided when the 10mg Pembro dose was the most effective on the basis of ORR, OS and PFS	-	-	-	OS:HR for pembrolizumab 2 mg/kg VS docetaxel: 0.71 (95% CI 0.58–0.88; p=0.0008). HR for pembrolizumab 10 mg/kg VS docetaxel: 0.61 (0.49–0.75; p<0.0001).

Table S9: Dose escalation Phase II/III trials (Additional characteristics)

1st author or ClinicalTrials.gov Identifier	Stratification factors	Statistical power for phase III	Reason for proceeding to phase III	Reason for not proceeding to phase III	Reason for trial termination	Reason for amendment	Primary endpoint result
Edelman	Duration of response to prior platinum therapy (relapse-free period <3 months vs ≥3 months)	360 deaths for an 80% power to detect a HR of 0.725 or a 2.3 month gain in median OS (from 6 to 8.3 months) (2-sided alpha=0.05)	Dinutuximab plus irinotecan was well tolerated with no unanticipated adverse events (AEs) when dinutuximab is given at a dose up to 16 mg/m2	-	-	-	HR for Dinutuximab+Irinotecan vs Irinotecan=1.12 (CI, 0.9,1.4,p=0.3132)<br>HR for Dinutuximab+Irinotecan vs Topotecan= 1.05 (CI, 0.8,1.4,p=0.7233)
Paz-Ares L	NR	NR	-	-	-	-	N/A
NCT04254471	NR	NR	-	-	-	-	N/A
NCT05001724	NR	NR	-	-	-	-	N/A

Table S10: Multi-Arm Multi Stage (MAMS) phase II/III trials (Additional characteristics)

1st author or ClinicalTrials.gov Identifier	Status	Countries	Centres	Histology	Stage	PS (Performance Status)	Median age (years)	Percentage males	Duration (Months)
Dziadziuszko	Ongoing	28	182	All	IIB,IV	ECOG 0-2	N/A	N/A	NR

Table S11: Multi-Arm Multi Stage (MAMS) phase II/III trials (Additional characteristics)

1st author or ClinicalTrials.gov Identifier	Stratification factors	Statistical power for phase III	Reason for proceeding to phase III	Reason for not proceeding to phase III	Reason for trial termination	Reason for amendment	Primary endpoint result
Dziadziuszko	NR	NR	-	-	-	-	N/A

Table S12: Trials with other design (Additional characteristics)									
1st author or ClinicalTrials.gov Identifier	Status	Countries	Centres	Histology	Stage	PS (Performance Status)	Median age (years)	Percentage males	Duration (Months)
Wislez	Completed only phase II portion	1	29	Nonsquamous	IIA,IIB,IIIA	ECOG 0-1	59,5	0,61	81 (April 2009-January 2016)
Besse	Completed only phase II portion	1	40	All types except Bronchioalveolar carcinoma	IA,IB	ECOG 0-1	59	60.5%	89 (November 2008-April 2016)
Govindan	Terminated	1	24	Squamous cell, adenocarcinoma, bronchoalveolar carcinoma, large cell anaplastic lung carcinoma	IIIB,IV	ECOG 0-1	NR	NR	35 (February 2013-June 2016)
NCT03653546	Terminated	1	NR	Squamous cell carcinoma	IV	ECOG 0-1	72.3	87.5%	28 (June 2014-October 2016)
NCT04206072	Ongoing	4	57	All types	IV	ECOG 0-1	NR	NR	N/A

Table S13: Trials with other design (Additional characteristics)							
1st author or ClinicalTrials.gov Identifier	Stratification factors	Statistical power for phase III	Reason for proceeding to phase III	Reason for not proceeding to phase III	Reason for trial termination	Reason for amendment	Primary endpoint result
Wislez	center, EGFR status (mutated vs wild type or unknown), ERCC1 expression level (positive vs negative or unknown), sex, disease stage (IIA vs IIA vs IIB).	150 patients with a power of 93%	-	Unreliability of the ERCC1 immunohistochemical readouts	-	-	Success rate of 80% (90% CI, 74.6% to 85.4%). The success rates in arms A and B were 77% (90% CI, 69.0% to 85.1%) and 83% (90% CI, 75.8% to 90.0%), respectively
Besse	stage (IA versus IB), histology (squamous versus non-squamous cell carcinoma)	80% as an acceptable compliance rate, with a statistical power of 90% and a type I error rate (one-sided) of 0.05.	-	Due to outcome of exploratory efficacy data and lack of optimal pazopanib dosing.	-	-	The compliance rates at 800 mg/day were 38% [95% CI: 23–55] in the pazopanib arm (39 patients) and 88% [73–96] in placebo arm (41 patients). Compliance rates at 400 mg/day were 69% [50–84] in pazopanib arm (32 patients) and 93% [77–99] in placebo arm (30 patients), giving a significant improvement in compliance between 800 and 400 mg/day in the pazopanib arm (P=0.027)
Govindan	NR	NR	-	-	NR	-	NR
NCT03653546	NR	NR	-	-	NR	-	NR
NCT04206072	NR	360 patients to provide a 86% power at a two-sided 5% significance level with an estimated 20% dropout rate	-	-	-	-	N/A

Table S14: Detailed Cochrane risk-of-bias tool for randomized trials (RoB 2) assessment					
**Unique ID**	LUX-LUNG 1	**Study ID**	NCT00656136	**Assessor**	Dionysios Palermos
**Ref or Label**		**Aim**	assignment to intervention (the 'intention-to-treat' effect)		
**Experimental**	Afatinib	**Comparator**	Placebo	**Source**	Journal article(s); Non-commercial trial registry record (e.g. ClinicalTrials.gov record)
**Outcome**	OS	**Results**	HR=1.08	**Weight**	1

**Domain**	**Signalling question**	**Response**	**Comments**
**Bias arising from the randomization process**	1.1 Was the allocation sequence random?	Y	The randomisation sequence was generated by an independent team from the trial sponsor with a validated computer system (clinical trial supply system).
	1.2 Was the allocation sequence concealed until participants were enrolled and assigned to interventions?	Y	
	1.3 Did baseline differences between intervention groups suggest a problem with the randomization process?	N	Baseline characteristics were much the same between the two groups
	**Risk of bias judgement**	**Low**	The randomisation sequence was generated by an independent team from the trial sponsor with a validated computer system (clinical trial supply system).

			Baseline characteristics were much the same between the two groups
**Bias due to deviations from intended interventions**	2.1.Were participants aware of their assigned intervention during the trial?	N	Investigators, patients, and the trial sponsor were masked to block size and treatment assignments.
	2.2.Were carers and people delivering the interventions aware of participants' assigned intervention during the trial?	N	
	2.3. If Y/PY/NI to 2.1 or 2.2: Were there deviations from the intended intervention that arose because of the experimental context?	NA	
	2.4 If Y/PY to 2.3: Were these deviations likely to have affected the outcome?	NA	
	2.5. If Y/PY/NI to 2.4: Were these deviations from intended intervention balanced between groups?	NA	
	2.6 Was an appropriate analysis used to estimate the effect of assignment to intervention?	Y	The primary endpoint was analysed on an intention-to-treat basis
	2.7 If N/PN/NI to 2.6: Was there potential for a substantial impact (on the result) of the failure to analyse participants in the group to which they were randomized?	NA	
	**Risk of bias judgement**	**Low**	Investigators, patients, and the trial sponsor were masked to block size and treatment assignments.

			The primary endpoint was analysed on an intention-to-treat basis
**Bias due to missing outcome data**	3.1 Were data for this outcome available for all, or nearly all, participants randomized?	Y	There was no evidence of a difference between treatment groups when analysed by subgroup
	3.2 If N/PN/NI to 3.1: Is there evidence that result was not biased by missing outcome data?	NA	
	3.3 If N/PN to 3.2: Could missingness in the outcome depend on its true value?	NA	
	3.4 If Y/PY/NI to 3.3: Is it likely that missingness in the outcome depended on its true value?	NA	
	**Risk of bias judgement**	**Low**	There was no evidence of a difference between treatment groups when analysed by subgroup
**Bias in measurement of the outcome**	4.1 Was the method of measuring the outcome inappropriate?	N	The key assumption that led to the selection of this<br><br>primary endpoint was that the survival in the control<br><br>group in this trial was expected to be short and similar to<br><br>the 4·7-month control median overall survival in the second-line and third-line non-small-cell lung cancer (NSCLC) phase 3 trial of erlotinib

	4.2 Could measurement or ascertainment of the outcome have differed between intervention groups?	N	By both independent and investigator assessment,<br><br>median progression-free survival was longer in the afatinib group than it was in the placebo
	4.3 Were outcome assessors aware of the intervention received by study participants?	N	Investigators, patients, and the trial sponsor were masked to block size<br><br>and treatment assignments.
	4.4 If Y/PY/NI to 4.3: Could assessment of the outcome have been influenced by knowledge of intervention received?	NA	
	4.5 If Y/PY/NI to 4.4: Is it likely that assessment of the outcome was influenced by knowledge of intervention received?	NA	
	**Risk of bias judgement**	**Low**	The key assumption that led to the selection of this<br><br>primary endpoint was that the survival in the control<br><br>group in this trial was expected to be short and similar to<br><br>the 4·7-month control median overall survival in the second-line and third-line non-small-cell lung cancer (NSCLC) phase 3 trial of erlotinib<br><br>By both independent and investigator assessment,<br><br>median progression-free survival was longer in the afatinib group than it was in the placebo<br><br>Investigators, patients, and the trial sponsor were masked to block size<br><br>and treatment assignments.

**Bias in selection of the reported result**	5.1 Were the data that produced this result analysed in accordance with a pre-specified analysis plan that was finalized before unblinded outcome data were available for analysis?	Y	The investigators interpreted the data independently.
	5.2 ... multiple eligible outcome measurements (e.g. scales, definitions, time points) within the outcome domain?	PN	
	5.3 ... multiple eligible analyses of the data?	PN	
	**Risk of bias judgement**	**Low**	The investigators interpreted the data independently.
**Overall bias**	**Risk of bias judgement**	**Low**	The randomisation sequence was generated by an independent team from the trial sponsor with a validated computer system (clinical trial supply system).<br>Baseline characteristics were much the same between the two groups<br>Investigators, patients, and the trial sponsor were masked to block size<br>and treatment assignments.

			The primary endpoint was analysed on an intention-to-treat basis<br><br>There was no evidence of a difference between treatment groups when analysed by subgroup<br><br>The key assumption that led to the selection of this<br><br>primary endpoint was that the survival in the control<br><br>group in this trial was expected to be short and similar to<br><br>the 4·7-month control median overall survival in the second-line and third-line non-small-cell lung cancer (NSCLC) phase 3 trial of erlotinib<br><br>By both independent and investigator assessment,<br><br>median progression-free survival was longer in the afatinib group than it was in the placebo

			Investigators, patients, and the trial sponsor were masked to block size and treatment assignments.<br><br>The investigators interpreted the data independently.

Unique ID	KEYNOTE-010	Study ID	NCT01905657	Assessor	Dionysios Palermos
Ref or Label		Aim	assignment to intervention (the 'intention-to-treat' effect)		
Experimental	Pembrolizumab	Comparator	Docetaxel	Source	Journal article(s); Statistical analysis plan (SAP); Non-commercial trial registry record (e.g. ClinicalTrials.gov record)
Outcome	OS, PFS	Results	Overall survival was significantly longer for	Weight	1

			pembrolizumab		

Domain	Signalling question	Response	Comments
**Bias arising from the randomization process**	1.1 Was the allocation sequence random?	Y	Patients were randomly assigned (1:1:1) with a central interactive voice-response system<br><br>The allocation schedule was generated by the system vendor<br><br>using a computerised randomised list generator
	1.2 Was the allocation sequence concealed until participants were enrolled and assigned to interventions?	Y	
	1.3 Did baseline differences between intervention groups suggest a problem with the randomization process?	N	Baseline characteristics were as expected for patients with advanced non-small-cell lung cancer and were balanced between groups
	**Risk of bias judgement**	**Low**	Patients were randomly assigned (1:1:1) with a central interactive voice-response system<br><br>The allocation schedule was generated by the system vendor<br><br>using a computerised randomised list generator<br><br>Baseline characteristics were as expected for patients with advanced non-small-cell lung cancer and were balanced between groups
**Bias due to deviations from intended interventions**	2.1.Were participants aware of their assigned intervention during the trial?	Y	We did this randomised, open-label, phase 2/3 study at 202 academic medical centres in 24 countries
	2.2.Were carers and people delivering the interventions aware of participants' assigned intervention during the trial?	Y	
	2.3. If Y/PY/NI to 2.1 or 2.2: Were there deviations from the intended intervention	Y	34 patients withdrew consent after learning they were allocated to the docetaxel group.

	that arose because of the experimental context?		
	2.4 If Y/PY to 2.3: Were these deviations likely to have affected the outcome?	PN	
	2.5. If Y/PY/NI to 2.4: Were these deviations from intended intervention balanced between groups?	NA	
	2.6 Was an appropriate analysis used to estimate the effect of assignment to intervention?	Y	
	2.7 If N/PN/NI to 2.6: Was there potential for a substantial impact (on the result) of the failure to analyse participants in the group to which they were randomized?	NA	
	**Risk of bias judgement**	**Some concerns**	We did this randomised, open-label, phase 2/3 study at 202 academic medical centres in 24 countries<br><br>34 patients withdrew consent after learning they were allocated to the docetaxel group.
**Bias due to missing outcome data**	3.1 Were data for this outcome available for all, or nearly all, participants randomized?	PY	
	3.2 If N/PN/NI to 3.1: Is there evidence that result was not biased by missing outcome data?	NA	

	3.3 If N/PN to 3.2: Could missingness in the outcome depend on its true value?	NA	
	3.4 If Y/PY/NI to 3.3: Is it likely that missingness in the outcome depended on its true value?	NA	
	**Risk of bias judgement**	**Low**	
**Bias in measurement of the outcome**	4.1 Was the method of measuring the outcome inappropriate?	N	the statistical analysis plan appropriately accounted for the multiple endpoints.
	4.2 Could measurement or ascertainment of the outcome have differed between intervention groups?	N	
	4.3 Were outcome assessors aware of the intervention received by study participants?	Y	Open label study
	4.4 If Y/PY/NI to 4.3: Could assessment of the outcome have been influenced by knowledge of intervention received?	PN	
	4.5 If Y/PY/NI to 4.4: Is it likely that assessment of the outcome was influenced by knowledge of intervention received?	NA	
	**Risk of bias judgement**	**Low**	the statistical analysis plan appropriately accounted for the multiple endpoints.<br><br>Open label study
**Bias in selection of**	5.1 Were the data that produced this result analysed in accordance with a pre-specified	Y	Two prespecified interim analyses were done by an unmasked statistician

**the reported result**	analysis plan that was finalized before unblinded outcome data were available for analysis?		
	5.2 ... multiple eligible outcome measurements (e.g. scales, definitions, time points) within the outcome domain?	PN	
	5.3 ... multiple eligible analyses of the data?	PN	
	**Risk of bias judgement**	**Low**	
**Overall bias**	**Risk of bias judgement**	**Some concerns**	Patients were randomly assigned (1:1:1) with a central interactive voice-response system<br><br>The allocation schedule was generated by the system vendor<br><br>using a computerised randomised list generator<br><br>Baseline characteristics were as expected for patients with advanced non-small-cell lung cancer and were balanced between groups<br><br>We did this randomised, open-label, phase 2/3 study at 202 academic medical centres in 24 countries<br><br>34 patients withdrew consent after learning they were allocated to the docetaxel group.

			the statistical analysis plan appropriately accounted for the multiple endpoints.<br><br>Open label study

Unique ID	BEVACIZUMAB	Study ID	NCT00021060	Assessor	Dionysios Palermos
**Ref or Label**		**Aim**	assignment to intervention (the 'intention-to-treat' effect)		
**Experimental**	Bevacizumab	**Comparator**	Paclitaxel-Carboplatin	**Source**	Journal article(s); Non-commercial trial registry record (e.g. ClinicalTrials.gov record); Personal communication with trialist

Outcome	OS	Results	HR=0.79 (95% CI, 0.67 to 0.92; P = 0.003)	Weight	1

Domain	Signalling question	Response	Comments
**Bias arising from the randomization process**	1.1 Was the allocation sequence random?	Y	Between July 2001 and April 2004, we conducted a randomized study in which 878 patients with recurrent or advanced non–small-cell lung cancer (stage IIIB or IV) were assigned to paclitaxel and carboplatin chemotherapy alone (paclitaxel–carboplatin group) (444 patients) or paclitaxel and carboplatin plus bevacizumab (paclitaxel–carboplat in–bevacizumab group) (434 patients).
	1.2 Was the allocation sequence concealed until participants were enrolled and assigned to interventions?	Y	
	1.3 Did baseline differences between intervention groups suggest a problem with the randomization process?	PY	The two groups were well balanced, except for a difference in distribution according to sex (men accounted for 58% of patients in the paclitaxel–carboplatin group and 50% of those in the paclitaxel–carboplatin–bevacizumab

			group; P = 0.03, with Fisher’s exact test)
	**Risk of bias judgement**	**Some concerns**	Between July 2001 and April 2004, we conducted a randomized study in which 878 patients with recurrent or advanced non–small-cell lung cancer (stage IIIB or IV) were assigned to paclitaxel and carboplatin chemotherapy alone (paclitaxel–carboplatin group) (444 patients) or paclitaxel and carboplatin plus bevacizumab (paclitaxel–carboplat in–bevacizumab group) (434 patients). The two groups were well balanced, except for a difference in distribution according to sex (men accounted for 58% of patients in the paclitaxel–carboplatin group and 50% of those in the paclitaxel–carboplatin–bevacizumab group; P = 0.03, with Fisher’s exact test)

**Bias due to deviations from intended intervention s**	2.1.Were participants aware of their assigned intervention during the trial?	Y	Open-label study
	2.2.Were carers and people delivering the interventions aware of participants' assigned intervention during the trial?	Y	
	2.3. If Y/PY/NI to 2.1 or 2.2: Were there deviations from the intended intervention that arose because of the experimental context?	NI	
	2.4 If Y/PY to 2.3: Were these deviations likely to have affected the outcome?	NA	
	2.5. If Y/PY/NI to 2.4: Were these deviations from intended intervention balanced between groups?	NA	
	2.6 Was an appropriate analysis used to estimate the effect of assignment to intervention?	PY	
	2.7 If N/PN/NI to 2.6: Was there potential for a substantial impact (on the result) of the failure to analyse participants in the group to which they were randomized?	NA	
	**Risk of bias judgement**	**Some concerns**	Open-label study
**Bias due to missing**	3.1 Were data for this outcome available for all, or nearly all, participants randomized?	Y	

**outcome data**	3.2 If N/PN/NI to 3.1: Is there evidence that result was not biased by missing outcome data?	NA	
	3.3 If N/PN to 3.2: Could missingness in the outcome depend on its true value?	NA	
	3.4 If Y/PY/NI to 3.3: Is it likely that missingness in the outcome depended on its true value?	NA	
	**Risk of bias judgement**	**Low**	
**Bias in measurement of the outcome**	4.1 Was the method of measuring the outcome inappropriate?	N	OS as the primary outcome
	4.2 Could measurement or ascertainment of the outcome have differed between intervention groups?	PN	
	4.3 Were outcome assessors aware of the intervention received by study participants?	Y	
	4.4 If Y/PY/NI to 4.3: Could assessment of the outcome have been influenced by knowledge of intervention received?	PN	
	4.5 If Y/PY/NI to 4.4: Is it likely that assessment of the outcome was influenced by knowledge of intervention received?	NA	
	**Risk of bias judgement**	**Low**	OS as the primary outcome

Bias in selection of the reported result	5.1 Were the data that produced this result analysed in accordance with a pre-specified analysis plan that was finalized before unblinded outcome data were available for analysis?	Y	After the second planned interim analysis, the independent data monitoring committee recommended the release of the study results in March 2005, since the criteria for significance prespecified in the protocol had been met (Wald statistic, 2.67; O'Brien–Fleming boundary, at 72.2% information, 2.41)
	5.2 ... multiple eligible outcome measurements (e.g. scales, definitions, time points) within the outcome domain?	N	
	5.3 ... multiple eligible analyses of the data?	PN	
	**Risk of bias judgement**	**Low**	After the second planned interim analysis, the independent data monitoring committee recommended the release of the study results in March 2005, since the criteria for significance prespecified in the protocol had been met (Wald statistic, 2.67; O'Brien–Fleming boundary, at 72.2%

			information, 2.41)
**Overall bias**	**Risk of bias judgement**	**Some concerns**	Between July 2001 and April 2004, we conducted a randomized study in which 878 patients with recurrent or advanced non–small-cell lung cancer (stage IIIB or IV) were assigned to paclitaxel and carboplatin chemotherapy alone (paclitaxel–carboplatin group) (444 patients) or paclitaxel and carboplatin plus bevacizumab (paclitaxel–carboplat in–bevacizumab group) (434 patients). The two groups were well balanced, except for a difference in distribution according to sex (men accounted for 58% of patients in the paclitaxel–carboplatin group and 50% of those in the paclitaxel–carboplatin–bevacizumab group; P = 0.03, with Fisher's exact test)

			Open-label study<br><br>Twenty-eight patients were excluded from the primary analysis because of eligibility violations or inadequate data (nine patients because of incorrect disease stage, six because of receipt of radiation therapy within three weeks before entry into the study, four because of histologic findings of squamous-cell cancer, and nine for other reasons)

			OS as the primary outcome<br><br>After the second planned interim analysis, the independent data monitoring committee recommended the release of the study results in March 2005, since the criteria for significance prespecified in the protocol had been met (Wald statistic, 2.67; O'Brien–Fleming boundary, at 72.2% information, 2.41)

**Unique ID**	MINNIE PEARL	**Study ID**	NCT00193362	**Assessor**	Dionysios Palermos
**Ref or Label**		**Aim**	assignment to intervention (the 'intention-to-treat' effect)		
**Experimental**	Gemcitabine-Vinorelbine	**Comparator**	Paclitaxel-Carboplatin-Gemcitabine-	**Source**	
**Outcome**	OS	**Results**	(P = 0.269 log rank)	**Weight**	1

Domain	Signalling question	Response	Comments
**Bias arising from the randomization process**	1.1 Was the allocation sequence random?	Y	Initially, in the phase II portion of the trial, patients were randomly allocated to 4 regimens, and the patients that were randomized to the PCG and gemcitabine/vinorelbine regimens formed the initial cohorts in the randomized phase III design. Subsequently, further patients were randomized to receive either PCG or gemcitabine/vinorelbine.
	1.2 Was the allocation sequence concealed until participants were enrolled and assigned to interventions?	PY	
	1.3 Did baseline differences between intervention groups suggest a problem with the randomization process?	PN	Patients were well matched in reference to age, sex, PS, and stage.

	**Risk of bias judgement**	**Low**	Initially, in the phase II portion of the trial, patients were randomly allocated to 4 regimens, and the patients that were randomized to the PCG and gemcitabine/vinorelbine regimens formed the initial cohorts in the randomized phase III design. Subsequently, further patients were randomized to receive either PCG or gemcitabine/vinorelbine. Patients were well matched in reference to age, sex, PS, and stage.
**Bias due to deviations from intended intervention s**	2.1.Were participants aware of their assigned intervention during the trial?	Y	Open label study
	2.2.Were carers and people delivering the interventions aware of participants' assigned intervention during the trial?	Y	
	2.3. If Y/PY/NI to 2.1 or 2.2: Were there deviations from the intended intervention that arose because of the experimental context?	PN	
	2.4 If Y/PY to 2.3: Were these deviations likely to have affected the outcome?	NA	
	2.5. If Y/PY/NI to 2.4: Were these deviations from intended intervention balanced between groups?	NA	
	2.6 Was an appropriate analysis used to estimate the effect of assignment to intervention?	PN	

	2.7 If N/PN/NI to 2.6: Was there potential for a substantial impact (on the result) of the failure to analyse participants in the group to which they were randomized?	PN	
	**Risk of bias judgement**	**Some concerns**	
**Bias due to missing outcome data**	3.1 Were data for this outcome available for all, or nearly all, participants randomized?	PY	
	3.2 If N/PN/NI to 3.1: Is there evidence that result was not biased by missing outcome data?	NA	
	3.3 If N/PN to 3.2: Could missingness in the outcome depend on its true value?	NA	
	3.4 If Y/PY/NI to 3.3: Is it likely that missingness in the outcome depended on its true value?	NA	
	**Risk of bias judgement**	**Low**	
**Bias in measurement of the outcome**	4.1 Was the method of measuring the outcome inappropriate?	N	Overall survival was calculated according to an intent-to-treat analysis of all patients randomly assigned
	4.2 Could measurement or ascertainment of the outcome have differed between intervention groups?	PN	

	4.3 Were outcome assessors aware of the intervention received by study participants?	Y	
	4.4 If Y/PY/NI to 4.3: Could assessment of the outcome have been influenced by knowledge of intervention received?	PN	
	4.5 If Y/PY/NI to 4.4: Is it likely that assessment of the outcome was influenced by knowledge of intervention received?	NA	
	**Risk of bias judgement**	**Low**	Overall survival was calculated according to an intent-to-treat analysis of all patients randomly assigned
**Bias in selection of the reported result**	5.1 Were the data that produced this result analysed in accordance with a pre-specified analysis plan that was finalized before unblinded outcome data were available for analysis?	Y	
	5.2 ... multiple eligible outcome measurements (e.g. scales, definitions, time points) within the outcome domain?	PN	
	5.3 ... multiple eligible analyses of the data?	PN	
	**Risk of bias judgement**	**Low**	
**Overall bias**	**Risk of bias judgement**	**Some concerns**	

**Unique ID**	IFCT-0802	**Study ID**	IFCT-0802	**Assessor**	Dionysios Palermos
**Ref or Label**		**Aim**	assignment to intervention (the 'intention-to-treat' effect)		
**Experimental**	Paclitaxel-Carboplatin-Gemcitabine	**Comparator**	Paclitaxel-Carboplatin	**Source**	
**Outcome**	RR	**Results**	(P = .032; HR 1.309; 95% CI: 1.03 to 1.67)	**Weight**	1

Domain	Signalling question	Response	Comments
**Bias arising from the randomization process**	1.1 Was the allocation sequence random?	Y	A total of 324 patients were randomly assigned to the two arms.
	1.2 Was the allocation sequence concealed until participants were enrolled and assigned to interventions?	Y	
	1.3 Did baseline differences between intervention groups suggest a problem with the randomization process?	PY	
	**Risk of bias judgement**	**Some concerns**	A total of 324 patients were randomly assigned to the two arms.

Bias due to deviations from intended intervention s	2.1.Were participants aware of their assigned intervention during the trial?	Y	Open label study
	2.2.Were carers and people delivering the interventions aware of participants' assigned intervention during the trial?	Y	
	2.3. If Y/PY/NI to 2.1 or 2.2: Were there deviations from the intended intervention that arose because of the experimental context?	PN	
	2.4 If Y/PY to 2.3: Were these deviations likely to have affected the outcome?	NA	
	2.5. If Y/PY/NI to 2.4: Were these deviations from intended intervention balanced between groups?	NA	
	2.6 Was an appropriate analysis used to estimate the effect of assignment to intervention?	NI	
	2.7 If N/PN/NI to 2.6: Was there potential for a substantial impact (on the result) of the failure to analyse participants in the group to which they were randomized?	PN	
	**Risk of bias judgement**	**Some concerns**	Open label study

**Bias due to missing outcome data**	3.1 Were data for this outcome available for all, or nearly all, participants randomized?	PN	16 patients with missing data
	3.2 If N/PN/NI to 3.1: Is there evidence that result was not biased by missing outcome data?	PY	Balance between the two cohorts regarding missing data
	3.3 If N/PN to 3.2: Could missingness in the outcome depend on its true value?	NA	
	3.4 If Y/PY/NI to 3.3: Is it likely that missingness in the outcome depended on its true value?	NA	
	**Risk of bias judgement**	**Low**	At least 13 patients with missing data<br><br>Balance between the two cohorts regarding missing data
**Bias in measurement of the outcome**	4.1 Was the method of measuring the outcome inappropriate?	N	RR was the main original end point. Statistical analyses followed the<br><br>principle of intention to treat
	4.2 Could measurement or ascertainment of the outcome have differed between intervention groups?	PN	
	4.3 Were outcome assessors aware of the intervention received by study participants?	Y	
	4.4 If Y/PY/NI to 4.3: Could assessment of the outcome have been influenced by knowledge of intervention received?	PY	
	4.5 If Y/PY/NI to 4.4: Is it likely that assessment of the outcome was influenced by knowledge of intervention received?	PN	

	**Risk of bias judgement**	**Some concerns**	RR was the main original end point. Statistical analyses followed the principle of intention to treat
**Bias in selection of the reported result**	5.1 Were the data that produced this result analysed in accordance with a pre-specified analysis plan that was finalized before unblinded outcome data were available for analysis?	PN	At the end of the planned randomization, the protocol was amended to further evaluate the impact of the two chemotherapy regimens on OS
	5.2 ... multiple eligible outcome measurements (e.g. scales, definitions, time points) within the outcome domain?	PN	
	5.3 ... multiple eligible analyses of the data?	PN	
	**Risk of bias judgement**	**Some concerns**	At the end of the planned randomization, the protocol was amended to further evaluate the impact of the two chemotherapy regimens on OS
**Overall bias**	**Risk of bias judgement**	**Some concerns**	A total of 324 patients were randomly assigned to the two arms.

			Open label study<br><br>At least 13 patients with missing data<br><br>Balance between the two cohorts regarding missing data<br><br>RR was the main original end point. Statistical analyses followed the principle of intention to treat

			At the end of the planned randomization, the protocol was<br><br>amended to further evaluate the impact of the two chemotherapy regimens on<br><br>OS

**Unique ID**	Group Study BR.18	**Study ID**	NCT00006229	**Assessor**	Dionysios Palermos
**Ref or Label**		**Aim**	assignment to intervention (the 'intention-to-treat' effect)		
**Experimental**	BMS-275291	**Comparator**	Placebo	**Source**	
**Outcome**	OS	**Results**	8.6 months for BMS-275291 versus 9.2 months for placebo for overall survival	**Weight**	1

Domain	Signalling question	Respons e	Comments
**Bias arising from the randomizati on process**	1.1 Was the allocation sequence random?	Y	Patients randomly assigned during the phase II portion of the study (n = 75) were included in the phase III analysis (planned prospectively) on an intent-to-treat basis in the primary analyses of time to progression and overall survival
	1.2 Was the allocation sequence concealed until participants were enrolled and assigned to interventions?	Y	
	1.3 Did baseline differences between intervention groups suggest a problem with the randomization process?	N	Baseline characteristics for the 774 patients are shown in Table 1, and were well balanced between the two treatment arms.
	**Risk of bias judgement**	**Low**	Patients randomly assigned during the phase II portion of the study (n = 75) were included in the phase III analysis (planned prospectively) on an intent-to-treat basis in the primary analyses of time to progression and overall survival Baseline characteristics for the 774 patients are shown in Table 1, and were well balanced between the two treatment arms.
**Bias due to deviations**	2.1.Were participants aware of their assigned intervention during the trial?	N	Triple masking (Participant, Care Provider, Investigator)

**from intended interventions**	2.2.Were carers and people delivering the interventions aware of participants' assigned intervention during the trial?	N	
	2.3. If Y/PY/NI to 2.1 or 2.2: Were there deviations from the intended intervention that arose because of the experimental context?	NA	
	2.4 If Y/PY to 2.3: Were these deviations likely to have affected the outcome?	NA	
	2.5. If Y/PY/NI to 2.4: Were these deviations from intended intervention balanced between groups?	NA	
	2.6 Was an appropriate analysis used to estimate the effect of assignment to intervention?	PY	
	2.7 If N/PN/NI to 2.6: Was there potential for a substantial impact (on the result) of the failure to analyse participants in the group to which they were randomized?	NA	
	**Risk of bias judgement**	**Low**	
**Bias due to missing outcome data**	3.1 Were data for this outcome available for all, or nearly all, participants randomized?	Y	All patients were assesed for the primary endpoint
	3.2 If N/PN/NI to 3.1: Is there evidence that result was not biased by missing outcome data?	NA	
	3.3 If N/PN to 3.2: Could missingness in the outcome depend on its true value?	NA	

	3.4 If Y/PY/NI to 3.3: Is it likely that missingness in the outcome depended on its true value?	NA	
	**Risk of bias judgement**	**Low**	
**Bias in measurement of the outcome**	4.1 Was the method of measuring the outcome inappropriate?	N	
	4.2 Could measurement or ascertainment of the outcome have differed between intervention groups?	N	
	4.3 Were outcome assessors aware of the intervention received by study participants?	N	
	4.4 If Y/PY/NI to 4.3: Could assessment of the outcome have been influenced by knowledge of intervention received?	NA	
	4.5 If Y/PY/NI to 4.4: Is it likely that assessment of the outcome was influenced by knowledge of intervention received?	NA	
	**Risk of bias judgement**	**Low**	
**Bias in selection of the reported result**	5.1 Were the data that produced this result analysed in accordance with a pre-specified analysis plan that was finalized before unblinded outcome data were available for analysis?	Y	

	5.2 ... multiple eligible outcome measurements (e.g. scales, definitions, time points) within the outcome domain?	N	
	5.3 ... multiple eligible analyses of the data?	PN	
	**Risk of bias judgement**	**Low**	
**Overall bias**	**Risk of bias judgement**	**Low**	

**Unique ID**	Dinutuximab	**Study ID**	NCT03098030	**Assessor**	Dionysios Palermos
**Ref or Label**		**Aim**	assignment to intervention (the 'intention-to-treat' effect)		
**Experimental**	Dinutuximab-Irinotecan	**Comparator**	Topotecan-Irinotecan	**Source**	Journal article(s); Non-commercial trial registry record (e.g. ClinicalTrials.gov record)
**Outcome**	OS	**Results**	HR=1.12 (95% CI: 0.90–1.40; p = 0.3132)	**Weight**	1
**Domain**	**Signalling question**			**Response**	**Comments**

**Bias arising from the randomization process**	1.1 Was the allocation sequence random?	Y	patients were randomized 2:2:1 to receive irinotecan (Group A), dinutuximab/irinotecan (Group B), or topotecan (Group C)
	1.2 Was the allocation sequence concealed until participants were enrolled and assigned to interventions?	Y	
	1.3 Did baseline differences between intervention groups suggest a problem with the randomization process?	N	baseline characteristics were similar across treatment groups
	**Risk of bias judgement**	**Low**	
**Bias due to deviations from intended interventions**	2.1.Were participants aware of their assigned intervention during the trial?	Y	open-label study
	2.2.Were carers and people delivering the interventions aware of participants' assigned intervention during the trial?	Y	
	2.3. If Y/PY/NI to 2.1 or 2.2: Were there deviations from the intended intervention that arose because of the experimental context?	PN	Deviations were similar across all treatment groups
	2.4 If Y/PY to 2.3: Were these deviations likely to have affected the outcome?	NA	
	2.5. If Y/PY/NI to 2.4: Were these deviations from intended intervention balanced between groups?	NA	
	2.6 Was an appropriate analysis used to estimate the effect of assignment to intervention?	PY	The primary analysis of OS was performed in the intention-to-treat (ITT) analysis
	2.7 If N/PN/NI to 2.6: Was there potential for a substantial impact (on the result) of the failure	NA	

	to analyse participants in the group to which they were randomized?		
	**Risk of bias judgement**	**Low**	
**Bias due to missing outcome data**	3.1 Were data for this outcome available for all, or nearly all, participants randomized?	PY	10 out of 471 patients withdrew consent
	3.2 If N/PN/NI to 3.1: Is there evidence that result was not biased by missing outcome data?	NA	
	3.3 If N/PN to 3.2: Could missingness in the outcome depend on its true value?	NA	
	3.4 If Y/PY/NI to 3.3: Is it likely that missingness in the outcome depended on its true value?	NA	
	**Risk of bias judgement**	**Low**	
**Bias in measurement of the outcome**	4.1 Was the method of measuring the outcome inappropriate?	N	Median OS and PFS in each treatment group and the corresponding<br><br>2-sided 95% CIs were estimated using the Kaplan-Meier method
	4.2 Could measurement or ascertainment of the outcome have differed between intervention groups?	N	OS as the primary endpoint
	4.3 Were outcome assessors aware of the intervention received by study participants?	Y	Open-label study
	4.4 If Y/PY/NI to 4.3: Could assessment of the outcome have been influenced by knowledge of intervention received?	PN	OS as the primary endpoint

	4.5 If Y/PY/NI to 4.4: Is it likely that assessment of the outcome was influenced by knowledge of intervention received?	NA	
	**Risk of bias judgement**	**Low**	
**Bias in selection of the reported result**	5.1 Were the data that produced this result analysed in accordance with a pre-specified analysis plan that was finalized before unblinded outcome data were available for analysis?	Y	
	5.2 ... multiple eligible outcome measurements (e.g. scales, definitions, time points) within the outcome domain?	PN	
	5.3 ... multiple eligible analyses of the data?	PN	
	**Risk of bias judgement**	**Low**	
**Overall bias**	**Risk of bias judgement**	**Low**	

**Unique ID**	JCOG1201	**Study ID**	UMIN000012605	**Assessor**	DP
**Ref or Label**		**Aim**	assignment to intervention		

			(the 'intention-to-treat' effect)		
**Experimental**	CI	**Comparator**	CERR/O	**Source**	
**Outcome**	HR for OS	**Results**	(HR, 0.848 (95% CI, 0.650-1.105)) (one-sided P=0.11)	**Weight**	1

Domain	Signalling question	Response	Comments
**Bias arising from the randomization process**	1.1 Was the allocation sequence random?	Y	Patients are randomized to either the CE arm or CI arm by the minimization method
	1.2 Was the allocation sequence concealed until participants were enrolled and assigned to interventions?	Y	
	1.3 Did baseline differences between intervention groups suggest a problem with the randomization process?	N	The characteristics of the patients were well balanced between CE arm and CI arm
	**Risk of bias judgement**	**Low**	Patients are randomized to either the CE arm or CI arm by the minimization method<br><br>The characteristics of the patients were well balanced between CE arm and CI arm
**Bias due to deviations from intended**	2.1.Were participants aware of their assigned intervention during the trial?	Y	Open label trial
	2.2.Were carers and people delivering the interventions aware of participants' assigned intervention during the trial?	Y	

**intervention s**	2.3. If Y/PY/NI to 2.1 or 2.2: Were there deviations from the intended intervention that arose because of the experimental context?	PN	
	2.4 If Y/PY to 2.3: Were these deviations likely to have affected the outcome?	NA	
	2.5. If Y/PY/NI to 2.4: Were these deviations from intended intervention balanced between groups?	NA	
	2.6 Was an appropriate analysis used to estimate the effect of assignment to intervention?	NI	
	2.7 If N/PN/NI to 2.6: Was there potential for a substantial impact (on the result) of the failure to analyse participants in the group to which they were randomized?	PN	
	**Risk of bias judgement**	**Some concerns**	
**Bias due to missing outcome data**	3.1 Were data for this outcome available for all, or nearly all, participants randomized?	NI	
	3.2 If N/PN/NI to 3.1: Is there evidence that result was not biased by missing outcome data?	PN	
	3.3 If N/PN to 3.2: Could missingness in the outcome depend on its true value?	N	
	3.4 If Y/PY/NI to 3.3: Is it likely that missingness in the outcome depended on its true value?	NA	

	**Risk of bias judgement**	**Low**	
**Bias in measurement of the outcome**	4.1 Was the method of measuring the outcome inappropriate?	N	RR, OS
	4.2 Could measurement or ascertainment of the outcome have differed between intervention groups?	PN	RR
	4.3 Were outcome assessors aware of the intervention received by study participants?	Y	Open label
	4.4 If Y/PY/NI to 4.3: Could assessment of the outcome have been influenced by knowledge of intervention received?	PY	RR phase 2 (Open label)
	4.5 If Y/PY/NI to 4.4: Is it likely that assessment of the outcome was influenced by knowledge of intervention received?	PN	
	**Risk of bias judgement**	**Some concerns**	
**Bias in selection of the reported result**	5.1 Were the data that produced this result analysed in accordance with a pre-specified analysis plan that was finalized before unblinded outcome data were available for analysis?	Y	Interim analysis for phase 2
	5.2 ... multiple eligible outcome measurements (e.g. scales, definitions, time points) within the outcome domain?	PN	
	5.3 ... multiple eligible analyses of the data?	PN	Just one interim analysis to proceed to phase 3

	**Risk of bias judgement**	**Low**	
**Overall bias**	**Risk of bias judgement**		
